# Automatic prediction of non-iodine-avid status in lung metastases for radioactive I^131^ treatment in differentiated thyroid cancer patients

**DOI:** 10.3389/fendo.2024.1429115

**Published:** 2024-06-11

**Authors:** Xinyi Gao, Haoyi Chen, Yun Wang, Feijia Xu, Anni Zhang, Yong Yang, Yajia Gu

**Affiliations:** ^1^ Shanghai Institute of Medical Imaging, Fenglin Road, Shanghai, China; ^2^ Department of Radiology, Fudan University Shanghai Cancer Center, Dongan Road, Shanghai, China; ^3^ Department of Radiology, Zhejiang Cancer Hospital, Banshan East Road, Hangzhou, Zhejiang, China; ^4^ Hangzhou Dianzi University, Baiyang, Qiantang, Hangzhou, Zhejiang, China; ^5^ Department of Nuclear medicine, Zhejiang Cancer Hospital, Banshan East Road, Hangzhou, Zhejiang, China; ^6^ Department of Radiology, Shanghai Tenth People’s Hospital, Tongji University, Shanghai, China; ^7^ Department of Radiology, The First People’s Hospital of Fuyang, Beihuan Road, Hangzhou, Zhejiang, China

**Keywords:** differentiated thyroid cancer, radioactive iodine therapy, lung metastases, RAI refractory, deep learning

## Abstract

**Objectives:**

The growing incidence of differentiated thyroid cancer (DTC) have been linked to insulin resistance and metabolic syndrome. The imperative need for developing effective diagnostic imaging tools to predict the non-iodine-avid status of lung metastasis (LMs) in differentiated thyroid cancer (DTC) patients is underscored to prevent unnecessary radioactive iodine treatment (RAI).

**Methods:**

Primary cohort consisted 1962 pretreated LMs of 496 consecutive DTC patients with pretreated initially diagnosed LMs who underwent chest CT and subsequent post-treatment radioiodine SPECT. After automatic lesion segmentation by SE V-Net, SE Net deep learning was trained to predict non-iodine-avid status of LMs. External validation cohort contained 123 pretreated LMs of 24 consecutive patients from other two hospitals. Stepwise validation was further performed according to the nodule’s largest diameter.

**Results:**

The SE-Net deep learning network yielded area under the receiver operating characteristic curve (AUC) values of 0.879 (95% confidence interval: 0.852–0.906) and 0.713 (95% confidence interval: 0.613–0.813) for internal and external validation. With the LM diameter decreasing from ≥10mm to ≤4mm, the AUCs remained relatively stable, for smallest nodules (≤4mm), the model yielded an AUC of 0.783. Decision curve analysis showed that most patients benefited using deep learning to decide radioactive I^131^ treatment.

**Conclusion:**

This study presents a noninvasive, less radioactive and fully automatic approach that can facilitate suitable DTC patient selection for RAI therapy of LMs. Further prospective multicenter studies with larger study cohorts and related metabolic factors should address the possibility of comprehensive clinical transformation.

## 1 Introduction

In recent decades, there has been a simultaneous rise in metabolic abnormalities, including chronic inflammation and insulin resistance, as well as an increase in the prevalence and incidence of thyroid cancer globally (1). The escalation of thyroid cancer may be linked to insulin resistance (2–4). Differentiated thyroid cancer (DTC) is the most common subtype, comprising approximately 95% of all cases (5, 6). Established risk factors for DTC include exposure to radiation, deficiencies or excesses in iodine, and the consumption of alcohol and tobacco. In recent studies, an increased prevalence of metabolic abnormalities, such as insulin resistance, obesity, hyperinsulinemia, and diabetes mellitus, has been associated with a higher incidence of differentiated thyroid cancer (7, 8).

Although most DTC carry with indolent clinical course and show good prognosis, metastatic DTC could be fatal, with 10% of patients developing distant metastasis (9, 10). Lung is the most common site for distant metastases in DTC, and lung metastases (LMs) has been reported accounting for 4% in adult DTC (11), with a higher risk in children and young adults, with an incidence rate up to 23% -25% (12, 13). The treatment of DTC typically involves total thyroidectomy followed by radioactive I^131^ therapy (14) (RAI). According to the iodine-concentrating status, LMs are classified as RAI-avid and RAI-refractory. RAI therapy is only recommended for patients with RAI-avid lung metastases. I^131^ therapy has shown limited efficacy in both the detection and treatment of RAI-refractory lung metastases. For those cases, the consideration of treatments with tyrosine kinase inhibitors are recommended. A timely shift in treatment strategy towards target therapy, followed by RAI therapy, may potentially reverse resistance to RAI in metastatic differentiated thyroid cancer (5, 15, 16).

The key challenge in utilizing RAI for DTC patients with LMs lies in the selection of patients who are likely to benefit from radioactive I^131^ and the exclusion of those with RAI-refractory LMs. The diagnosis of RAI-refractory lung metastases was mainly relied on diagnostic radioiodine whole-body scanning (DxWBS), which assesses the uptake of radioactive I^131^ in LMs prior to I^131^ therapy. However, the utilization of DxWBS has significantly declined over the past two decades (17), due to concerns regarding the potential short-term morbidity (18) and risk of secondary malignancy (19), especially in pediatric and young patients (20) with metastatic DTC. Currently in China, DxWBS is not routinely conducted prior to RAI treatment for patients with distant metastatic DTC. Following clinical diagnosis of LMs post-thyroidectomy, DTC patients are typically treated with radioactive iodine and undergo post-treatment-WBS (RxWBS). However, in cases of RAI-refractory LMs, patients may receive unnecessarily high doses of radioactive I^131^, presenting a significant clinical challenge. Both diagnostic (DxWBS) and post-treatment (RxWBS) scans are unable to accurately predict iodine uptake in lung metastases without the administration of radioactive I^131^, potentially leading to unnecessary radioactive accumulation and treatment delay in DTC patients with RAI-refractory LMs. The imperative need for developing effective diagnostic imaging tools to predict the non-iodine-avid status of lung metastasis in DTC is underscored in order to prevent unnecessary radioactive iodine treatment and tailor effective therapeutic interventions, such as targeted therapy, for individual patients.

The American Thyroid Association Guidelines (14) recommended the use of chest CT for identifying RAI-refractory DTC in routine clinical practice. However, actually distinguishing the initial iodine-concentrating status of DTC lung metastases on CT scans poses a challenge for clinicians. Radiology artificial intelligence enables the extraction of image-driven biomarkers for the objective in-depth classification of tumor phenotypes (21) and their microenvironment in DTC patients. However, the feasibility of artificial intelligence utilizing routine chest CT scans to predict non-iodine-avid LMs from iodine-avid LMs remains to be determined. The identification of CT-driven biomarkers may facilitate the noninvasive classification of non-iodine-avid status of LMs in DTC patients, thereby assisting in timely clinical decision-making for selecting patients most likely to benefit from RAI therapy.

It is an urgent clinical problem to predict the initial I^131^ uptake of lung metastases in patients with differentiated thyroid cancer without introducing any radionuclides, so as to predict the efficacy of radioactive I^131^ therapy. In this research, it was hypothesized that distinct image-driven biomarkers present in non-contrast chest CT scans prior to radioactive iodine treatment could indicate varying levels of tumor heterogeneity between noniodine-avid and iodine-avid lung metastases in patients with differentiated thyroid cancer. To test this hypothesis, an automated and noninvasive approach utilizing two deep learning networks was developed to differentiate RAI-refractory lung metastases from RAI-avid lung metastases in differentiated thyroid cancer patients based on pre-treatment non-contrast chest CT scans. To assess the generalizability of the models, internal and external validations were conducted across multiple medical centers.

## 2 Materials and methods

### 2.1 Study design and participant

This observational study was in accordance with the Declaration of Helsinki and obtained institutional review board (IRB) approval of the Ethics Committee of Zhejiang Cancer Hospital (IRB-2022149), with written informed consent waived according to the rules. External validation was conducted retrospectively from data obtained for clinical purposes, and the Ethics Committee of Fudan University Shanghai Cancer Center and the Ethics Committee of Shanghai Tenth People’s Hospital have confirmed that no ethical approval was required. The STROBE guideline was used for reporting in this study.

**Figure 1:**
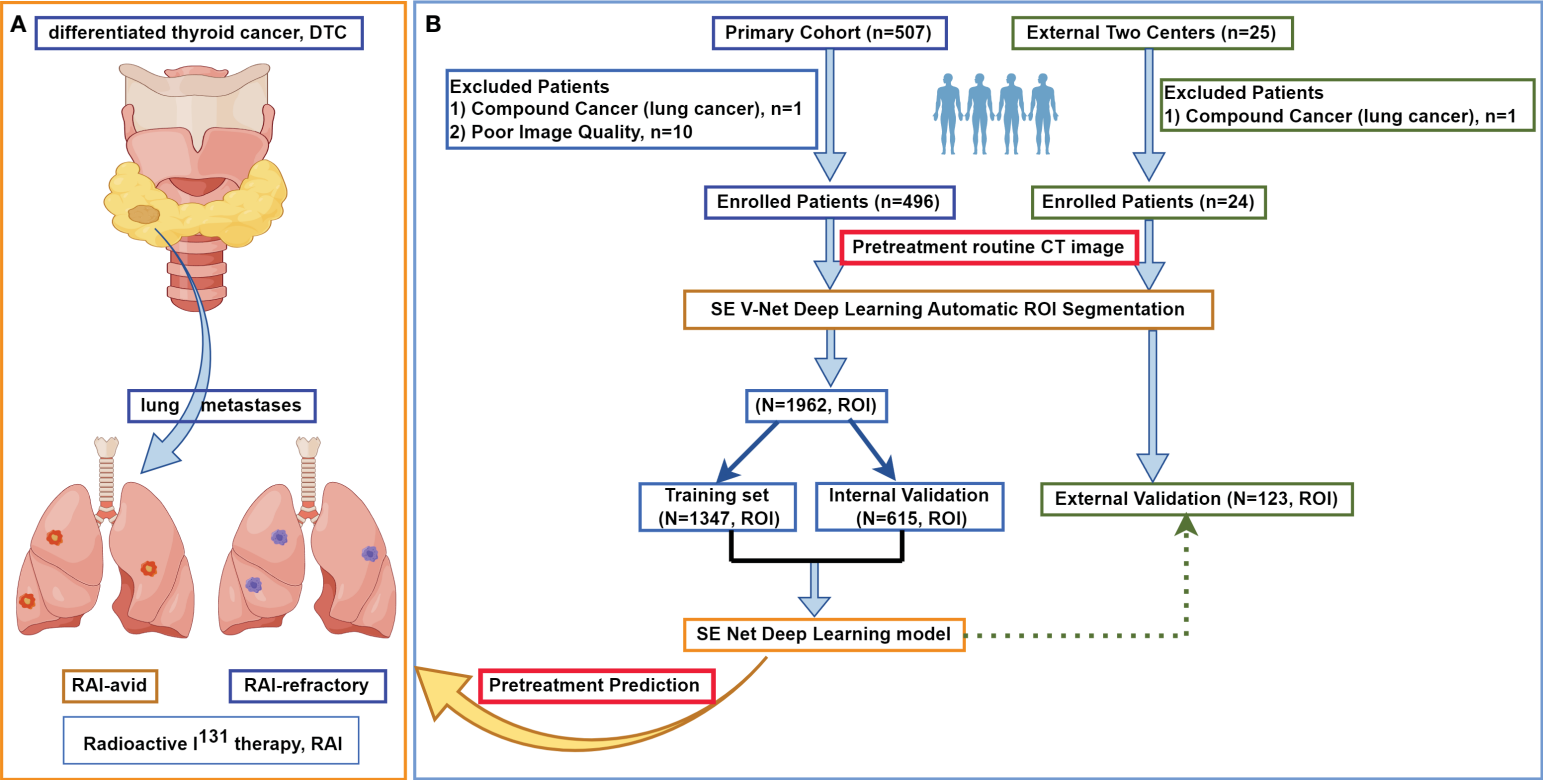
The flowchart of this study. **(A)** shows the research mechanism diagram of this study. **(B)** shows the study flowchart. In this multicenter study, a total of 520 patients with differentiated thyroid cancer treated at three large medical centers in China were included, with a collective total of 2085 lung metastases. After automatic lesion segmentation by SE V-Net, SE Net deep learning was trained to predict non-iodine-avid status of LMs. ROI, region of interest; RAI, radioactive I^131^ treatment; DTC, differentiated thyroid cancer.

**Figure 1** (by Figdraw) shows the flowchart of this study. The medical records of 496 consecutive DTC patients with pretreated initially diagnosed LMs who underwent both chest CT and subsequent RxWBS single-photon emission computed tomography (SPECT) between February 2009 and October 2022 admitted in the nuclear medicine ward of Zhejiang Cancer Hospital were reviewed. The CT images and subsequent RxWBS SPECT scans at the time of initial LMs diagnosis were independently reviewed by a nuclear medicine physician (YW, with 9 years of experience) and rechecked by a nuclear medicine professor (more than 20-year experience in the diagnosis and treatment of DTC LMs). LMs were classified as either RAI-avid or RAI-refractory according to the I^131^ uptake on WBS: LMs with clear uptake on WBS were defined as RAI-avid and LMs without visible or with <10% uptakes were defined as RAI refractory (22–24). The inclusion criteria were: 1) a histopathologically confirmed DTC; 2) performance of chest CT scans when LMs was first appeared; 3) with pretreated LMs diagnosed by following histopathology or RxWBS SPECT (RAI-avid); 4) For RAI-refractory LMs we applied more strict criteria than common clinical diagnosis: a) with <10% uptake on RxWBS or b) LMs without visible uptake on RxWBS SPECT and with a pathological conformation or c) LMs without visible uptake on RxWBS SPECT and with increasing thyroglobulin level and increasing in size of 6-month to 2-years follow-up by CT (in such case: RxWBS SPECT, medical records including serial thyroglobulin levels and serial CT scans would be rechecked through the multidisciplinary team composed of two radiologists and two nuclear medicine physicians, consistent diagnosis was made to determine RAI-refractory LMs), which satisfied diagnostic criteria of metastatic pulmonary disease (25); 5) availability of RxWBS SPECT scans with an interval of no more than two weeks between CT and subsequent RxWBS SPECT; 6) did not have cancer history other than DTC. Exclusion criteria included: 1) RAI treatment for LMs prior to initial LMs CT scans; 2) presence of compound cancer history; 3) unavailable of CT, RxWBS SPECT or medical data; 4) poor image quality that may hinder further analysis.

All the models were applied at per lesion level. In the primary cohort, for those patients with multiple LMs, only ≤ 5 lesions were randomly selected in further analysis. The detailed CT imaging parameters are listed in **Supplementary A**. Independent external validation cohort contained 24 consecutive DTC patients with pretreated LMs between October 2015 and October 2023 from Fudan University Shanghai Cancer Center (3 females, 6 males) and Shanghai Tenth People’s Hospital (11 females, 4 males). For those patients with multiple LMs in external validation cohorts, all lesions were selected in further analysis.

### 2.2 Image preprocessing and automatic ROI segmentation

**Figure 2:**
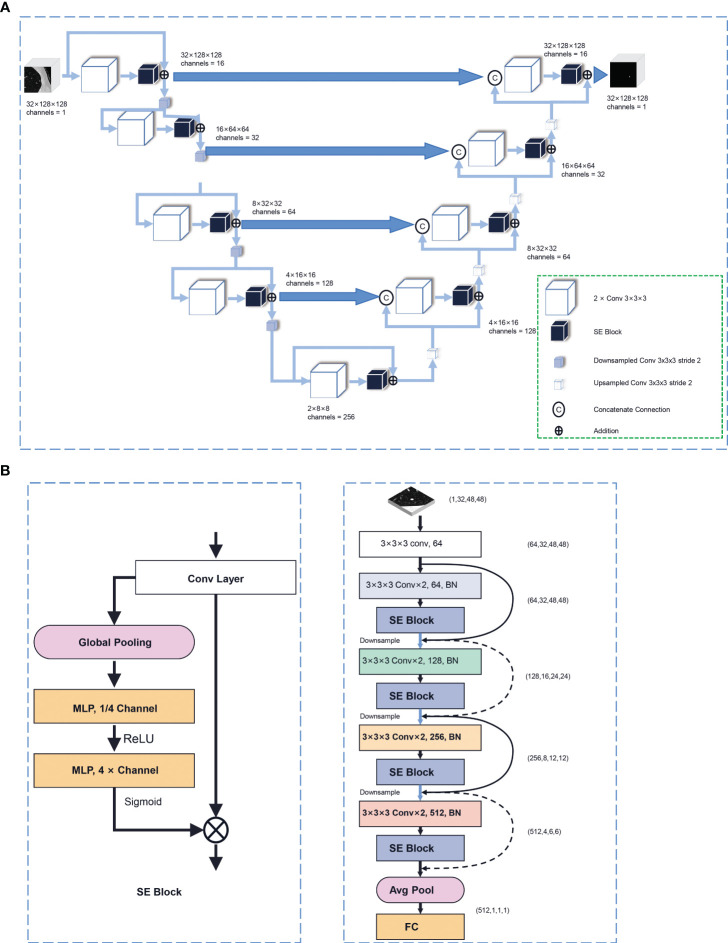
The architecture of our deep learning networks. **(A)** The pipeline of the automatic ROI segmentation network based on SE V-Net deep learning in this study. **(B)** Details of a SE Block and the pipeline of the deep learning network in this study based on SE Net.

CT scans were resampled into 1mm*1mm*1mm and were normalized into [0, 1]. Previously, we developed an automatic region of interest (ROI) segmentation network based on SE V-Net for DTC LMs. **Figure 2A** depicts the pipeline of this deep learning network. According to the chronological order, all the LMs of the 1st-187th DTC patients were manually segmented. A total of 1275 LMs were segmented on CT images by a skilled radiologist (ANZ), including 1034 subcentimeter LMs and 241 larger LMs. The delineations were then validated by a professor (YJG) and served as the ground truth for the study. The ROIs were subsequently partitioned into training (932) and validation (343) sets to construct the segmentation network. The dice coefficient between manual and automatic segmentation was evaluated. All CT images were input into this model for automatic segmentation and then reviewed by a radiologist (XYG.) to define the final ROI.

### 2.3 SE Net deep learning

Anaconda (Conda 4.13.0, Python 3.9.7, Continuum Analytics Inc., Austin, TX, USA) was used to build the model. **Figure 2B** demonstrates the pipeline of the deep learning network in this study based on SE Net. This model was constructed based on a combination of SE and Res modules. Initially, a batch containing a 3D image of dimensions (1, 32, 48, 48) was inputted into the model. Given that the CT image was monochromatic, the number of channels utilized was 1. The input layer consisted of 64 channels, followed by the subsequent residual module with SE module. After two 3 × 3 × 3 convolution layers and a SE module, the downsampling with 2 × 2 maximum pool was performed and sent to the next residual module after being directly added to the original input. The next residual module doubled the number of characteristic channels to 128. After a total of 4 residual modules, a Batch composed of (512) 3D images was output. After average pool compression, the global space was output to the full connection layer to get a probabilistic result. Among them, SE-Block was the key compression excitation module, which can compress the output results of the convolution layer in the global space, form a sequence of channel weights, learn the interdependent relationship between channels, re-calibrate the weights of each characteristic channel, and make the network pay attention to the channels with larger weights. The original feature map output of the convolution layer was first compressed in the global space to form a channel weight sequence, and then the weight of each characteristic channel was re-calibrated through two layers of linear layers. In the first layer, the number of characteristic channels was compressed to 1 stroke 4, and after ReLU activation function, the number of channels was extended to the original size and each weight value was mapped to 0–1 by Sigmoid activation function. Then the filtered channel weight sequence was multiplied by the original feature graph output of the convolution layer to achieve the redistribution of the channel weight.

### 2.4 Statistical analysis

The area under the receiver operating characteristic curve (AUC) with 95% confidential interval (CI) and accuracy (ACC), specificity, sensitivity, negative predictive value (NPV) and positive predictive value (PPV) were calculated to evaluate discrimination performance. Statistical analysis was performed using Anaconda (Conda 4.13.0, Python 3.9.7, Continuum Analytics Inc., Austin, TX, USA). The Chi-square test was used to compare categorical variables between subsets, and the independent-samples t-test was used to compare continuous variables. A p-value < 0.05 indicated a statistically significant difference.

## 3 Results

### 3.1 Patients characteristics

**Table 1** shows the characteristics of DTC patients with lung metastases. Statistical analysis indicated that there was no statistically significant difference (P=0.08) in sex between initial RAI-avid group and initial RAI-refractory group in the primary cohort. The age of patients in RAI-refractory group was found to be significantly older than that in RAI-avid group (P <0.001). The median largest diameter of RAI-avid group was 8.50 ± 5.68 mm. The median largest diameter of RAI-refractory group was 8.87 ± 4.35mm. There was no significant difference in the overall diameter between RAI-avid and RAI-refractory groups (P=0.06). The distribution of the largest diameter for both RAI-avid and RAI-refractory groups in the primary cohort are shown in **Supplementary B**. The segmented LMs were randomly assigned to the training group and the validation group at a ratio of 7:3. In the training group, 733 lung metastatic lesions (54.42%) were classified as RAI-avid lung metastases, while 614 lung metastatic lesions (45.58%) were classified as RAI-refractory lung metastases. In the internal validation group, 344 (55.93%) lung metastases exhibited RAI-avid characteristics, while 271 (44.07%) were classified as RAI-refractory lung metastases. There was no statistical difference in the diameter distribution within the training and internal validation groups (P=0.30). The detailed patient characteristics of the external validation cohorts from Fudan University Shanghai Cancer Center and Shanghai Tenth People’s Hospital were presented in **Table 1**. In external cohorts, there were 11 patients with 40 RAI-avid LMs and 13 patients with 83 RAI-refractory LMs. The nodule sizes in the external cohorts were significantly smaller than those in the primary cohort (P <0.001).

**Table 1:** Clinical characteristics in the primary cohort and external cohorts.

	Primary Cohort (n=496)		External Cohorts (n=24)	
	RAI-avid n=318	RAI-refractory n=178	RAI-avid n=11	RAI-refractory n=13
Sex				
Male	104 (32.70%)	72 (40.45%)	5 (45.45%)	5 (38.46%)
Female	214 (67.30%)	106 (59.55%)	6 (54.55%)	8 (61.54%)
Age, years	49.24 ± 15.90	54.93 ± 16.31	52.3 ± 19.72	49.15 ± 12.31
Max diameter, mm	8.50 ± 5.68	8.87 ± 4.35	6.40 ± 2.45	6.83 ± 2.52

RAI, radioactive I^131^ therapy.

### 3.2 Segmentation performance

The Dice Loss was used to evaluate the similarity between predictive segmentation mask (Predicted Mask) and real segmented image (Ground Truth), with the relationship defined as Dice Loss=1-Dice Coefficient. In this study, Dice loss served as the loss function for training SE V-Net, utilizing the Adam optimization algorithm. The learning rate was set at 0.01 for a total of 320 rounds of training. The Dice coefficient and Dice loss values for each round (Each Epoch) in the training set are presented in **Supplementary C**. The Dice coefficient exhibited rapid improvement in the first 20 rounds of training, followed by continuous fluctuations until the model achieved stability around 250th round. Upon completion of training in the final 320th round, the Dice coefficient for the validation set reached 0.821, with a corresponding Dice Loss of 0.179, demonstrating the successful implementation of an automatic ROI segmentation approach. Through this automatic deep learning method, the nodule region in the image can be segmented quickly, and then the independent nodule can be obtained by detecting the connected domain.

### 3.3 Prediction performance of overall nodule size

**Figure 3:**
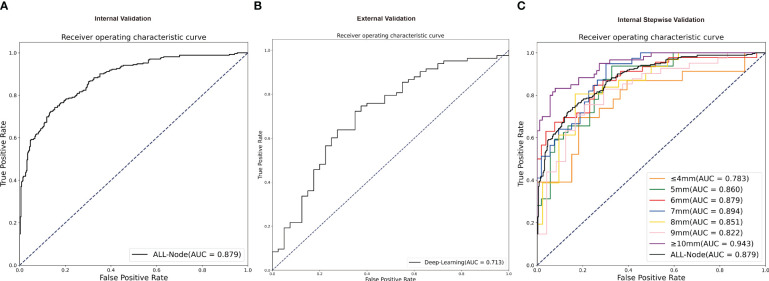
The receiver operating characteristic curves of validation groups. **(A)** The receiver operating characteristic curve of all nodules in independent internal validation. **(B)** The receiver operating characteristic curve of all nodules in independent external validation. **(C)** The receiver operating characteristic curve in stepwise internal validation of different nodule sizes. AUC, area under the receiver operating characteristic curve.

The AUC curves in internal and external validation were depicted in **Figure 3**, while **Table 2** outlined the detailed diagnostic performance of all nodule sizes in the internal validation. The AUC values for internal validation were 0.879 (95% CI: 0.852–0.906) and 0.713 (95% CI: 0.613–0.813). The accuracy rates for internal and external validation were 78.86% and 69.11%, respectively, with the sensitivity values of 0.768 and 0.723, and the specificity values of 0.805 and 0.625, respectively. The positive predictive values for internal and external validation were 0.756 and 0.800, respectively, while the negative predictive values were 0.815 and 0.721.

**Table 2:** Deep learning diagnosis performance in internal validation.

	Accuracy	AUC	Sensitivity	Specificity	PPV	NPV
All	78.86%	0.879	0.768	0.805	0.756	0.815
≥10mm	85.14%	0.943	0.833	0.864	0.806	0.884
9mm	78.46%	0.822	0.829	0.708	0.829	0.708
8mm	75.34%	0.851	0.806	0.714	0.676	0.833
7mm	79.12%	0.894	0.816	0.774	0.721	0.854
6mm	76.53%	0.879	0.696	0.827	0.780	0.754
5mm	76.19%	0.860	0.656	0.827	0.700	0.796
≤4mm	75.00%	0.783	0.652	0.818	0.714	0.771

AUC, area under the receiver operating characteristic curve; PPV, positive predictive value; NPV, negative predictive value.

**Figure 4:**
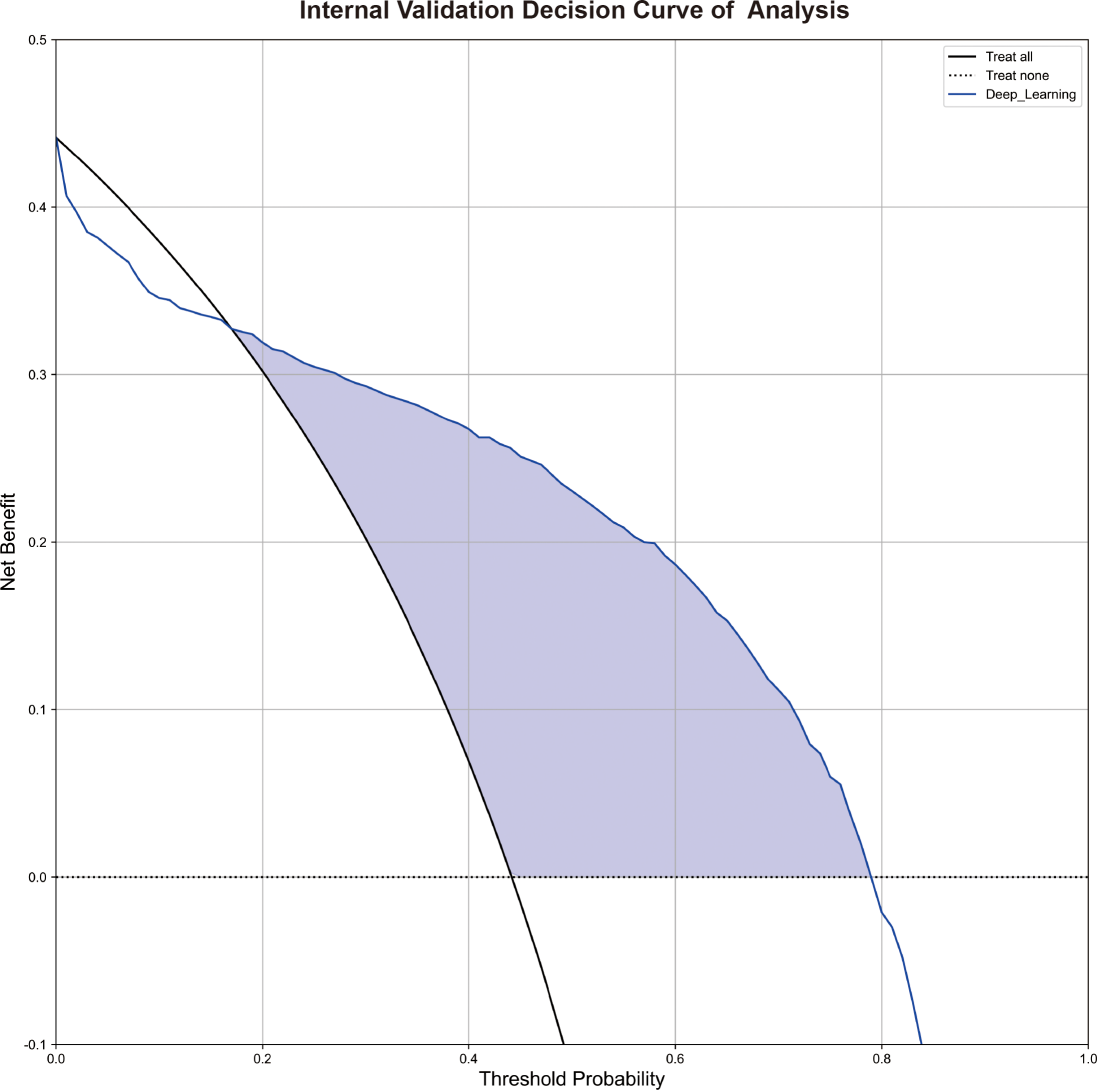
The decision curve analyses of the deep learning model in the internal validation. **Figure 4** shows the decision curve analyses of the SE Net deep learning in the internal validation.

The decision curve analysis (DCA) depicted in **Figure 4** indicated that within a large range of high-risk probabilities, the utilization of deep learning diagnosis based on non-contrast pretreatment CT scans for determining radioactive I^131^ treatment decisions for differentiated thyroid cancer patients with lung metastases yielded a greater clinical benefit compared to the strategies of treating all or treating none in the internal validation group. **Supplementary D** shows the representing CT images of a RAI-avid LM and a RAI-refractory LM and the heat maps of the features for the SE Net deep learning model.

### 3.4 Stepwise validation of different subgroups in the internal validation

Despite the absence of a significant discrepancy in the overall diameter between RAI-avid and RAI-refractory groups, diagnosing smaller lung metastases proves to be more challenging in clinical practice. Furthermore, the AUC value of the external validation was lower than that of the internal validation. On one hand, the sample of the external validation was smaller than the internal validation; on the other hand, the nodule size of external cohorts was significantly smaller than that in the primary cohort. To investigate the impact of smaller nodule size on the model’s predictive performance, we conducted stepwise validation based on the sizes of the lung metastatic nodules. These nodules were further divided into seven subgroups by the largest diameter as follows: ≥10mm, among 9mm, 8mm, 7mm, 6mm, 5mm, ≤ 4mm. Overall, the ≥10mm subgroup showed a higher likelihood of accurate prediction by deep learning model; and the AUCs remained relatively stable for 5mm to 9mm subgroups, but decreased for ≤ 4mm subgroup (**Figure 3**). For the ≤ 4mm subgroup, the SE-Net deep learning network achieved an AUC value of 0.783 for internal validation; the accuracy, sensitivity, specificity, positive predictive value, and negative predictive value were 75.00%, 0.652, 0.818, 0.714, and 0.771, respectively.

## 4 Discussion

The rising occurrence of DTC has been attributed to insulin resistance and metabolic syndrome (26). To predict I^131^ avidity noninvasively and with less radioactivity before radioactive I^131^ therapy treatment is crucial for the differentiated thyroid cancer patient with lung metastases. This study utilized a noninvasive and fully automatic deep learning approach based on pretreatment non-contrast chest CT scans to predict the iodine-avid status of lung metastatic nodules of radioactive I^131^ therapy treatment in differentiated thyroid cancer patients. The deep learning networks demonstrated robustness in both internal and external validation processes. Stepwise validation indicated the model’s stability even as the lung metastatic nodule largest diameter decreased from 9mm to 5mm. This study represents a novel demonstration of fully automatic, noninvasive and less radioactive approach that may aid DTC patient selection for lung metastases of radioactive I^131^ and identify patients with the RAI-refractory LMs before treatment to avoid unnecessary radiation and proper treatment delay, thus assisting clinical decision-making.

A notable proportion of fatalities among patients with differentiated thyroid cancer were attributed to secondary malignancies. In a longitudinal study spanning 30–50 years and involving 215 individuals diagnosed with thyroid cancer before the age of 21, the primary cause of death (68%) was identified as secondary cancer, with 73% of deceased patients^(19)^ having received cumulative doses of radioactive I^131^. In accordance with established principles of radioprotection (27), it is imperative to limit unnecessary radiation exposure in order to minimize the use of radioactive agents, especially in cases where minimal therapeutic benefit is anticipated. However, DTC patients with initial radioiodine-refractory lung metastases would not derive benefit from radioactive I^131^ therapy (28). Therefore, the use of less radioactive diagnostic methods is necessary to accurately predict the iodine-avid status of lung metastases in differentiated thyroid cancer, allowing for the assessment of treatment strategies in advance.

RAI-refractory cells in lung metastases of differentiated thyroid cancer patients may lose their ability to uptake iodine, but PET/CT tended to show positive uptake on fluorodeoxyglucose (29). Nakajoet al. observed a sensitivity, specificity, and accuracy of 45% (33 out of 73 lesions), 100% (207 out of 207 lesions), and 85.7% (240 out of 280 lesions) for distant metastases in differentiated thyroid cancer, with the low sensitivity potentially attributed to the limited resolution of PET for small lung metastases (30). Considering the scanning time, cost and availability, the routine PET/CT in most cases was unsatisfactory. Agate et.al (31) proposed that for the detection of lung metastases in DTC patients, chest examination using CT is preferable to PET/CT due to its superior spatial resolution in visualizing lung nodules. Additionally, the radiation dose associated with chest CT is lower comparing with DxWBS, RxWBS or PET/SPECT. Given the widespread availability and routine use of chest CT, the deep learning model developed in our study could serve as a viable alternative for monitoring the progression of lung metastases in DTC patients during longitudinal RAI treatment, offering greater efficacy than PET/CT while simultaneously reducing radiation exposure.

Age, sex, and tumor size are established prognostic factors in differentiated thyroid cancer. Previous studies indicated that patients over 55 years have a poorer response to RAI therapy (32) and a worse overall prognosis (33) in thyroid cancer with lung metastases, with mortality rates increasing with age (34). Consistent with these findings, the average age of patients in RAI-refractory group were 5 years older (P <0.05) than those in RAI-avid group in the primary cohort. Additionally, male gender has been associated with a worse prognosis compared to female gender. Moreover, the RAI-refractory group patients were older and had larger lung metastatic nodules in our study (22, 23, 35, 36).

Previous research has indicated that smaller lesions, specifically those less than 1cm in size, are associated with a more favorable prognosis in patients with metastatic differentiated thyroid cancer. A recent study highlighted the significance of the initial size of these metastatic lesions as a key predictor of prognosis and survival. Additionally, RAI therapy was more effective in treating smaller lesions compared to larger lesions. The median largest diameter of RAI-avid group was 8.50 ± 5.68mm, while the median largest diameter of RAI-refractory group was 8.87 ± 4.35mm. However, statistical analysis indicated that distinguishing iodine uptake based solely on the largest diameter of lung metastatic nodules was not feasible (P>0.05). Smaller lung metastases were found to be more challenging to diagnose compared to larger lung metastases. The AUC value for external validation was lower than that of internal validation, potentially due to the limited sample size and smaller nodule size of the external validation cohort. Additionally, the nodules in external cohorts were significantly smaller than those in the primary cohort (P<0.01). Furthermore, stepwise validation in the primary cohort further demonstrated that lung metastases measuring greater than 10mm were more accurately predicted by deep learning model. For those size ranging from 5mm to 9mm, the AUC remained relatively consistent, while for those measuring 4mm or less, the AUC decreased. In summary, the fully automated non-invasive deep learning model, utilizing pretreatment non-contrast chest CT scans, can effectively predict non-iodine-avid lung metastases across a range of sizes, including those with very small diameters (≤ 4mm).

Our research demonstrates that CT image-driven biomarkers can differentiate between RAI-avid and RAI-refractory lung metastases in patients with DTC, indicating tumor heterogeneity. Furthermore, abnormal glucose metabolism including DM, hyperinsulinemia and insulin resistance, were associated with the aggressiveness of DTC (37). Therefore, further study is needed to improve current understanding of inner mechanisms suggesting the relationship between metabolic abnormalities and RAI-refractory of radioactive I^131^ therapy in patients with DTC LMs. Importantly, our study provided a fully automated tool for predicting non-iodine-avid status of LMs in DTC noninvasively using CT image-driven biomarkers. RAI-refractory represents a major obstacle to the effective treatment for DTC patients with LMs. Understanding the mechanisms underlying RAI-refractory may lead to the development of novel treatment strategies for patient management and long-term survival. Future research should investigate the potential impact of insulin resistance on the response to radioactive I^131^ therapy in metastatic DTC. For instance, the triglyceride glucose index (TyG) is a novel and quantitative biomarker of insulin resistance (38), which may predict papillary thyroid cancer incidence (26). Type 2 diabetes was an independent risk factor for thyroid cancer specific death (39). In addition to CT imaging biomarkers, more potential biomarkers that may predict iodine-avid status and RAI-refractory need to be explored from a metabolic point of view. Insulin resistance plays a core role in the disease development of thyroid cancer, and repairing insulin resistance could improve the therapeutic effect (40). Further identification of modifiable metabolic factors affecting DTC lung metastases prognosis has the potential to improve radioactive I^131^ therapy treatment and patients’ outcomes.

This study has several limitations. First, although patients were enrolled from three of the largest nuclear medicine treatment centers for thyroid cancer in China the study population was relatively limited. Further multicenter studies with larger study cohorts covering diverse races, and metabolic factors should address the possibility of comprehensive clinical transformation. Second, selection bias may exist due to retrospective study design. Third, the imbalance between non-iodine-avid and iodine-avid status due to different incidence rates and limited sample size (in particular, the smaller sample of external validation) may introduce potential bias. Conducting future studies will significantly improve the quality of the content, fostering a stronger research community.

## 5 Conclusions

In conclusion, this multicenter study highlights a possible role of deep learning approach from routine pretreatment chest CT as an automatic, noninvasive, less radioactive and cost-effective way to predict non-iodine-avid status of LMs in DTC. This research has the potential to inform the optimal approach to aid the selection of DTC patients who are likely to benefit from radioactive I^131^ for initial lung metastases, and identify patients with RAI-refractory lung metastases to prevent unnecessary radioactive iodine treatment and tailor effective therapeutic interventions, ultimately improving personalized treatment strategies and patient outcomes.

## Data availability statement

The original contributions presented in the study are included in the article/**Supplementary Material**. Further inquiries can be directed to the corresponding author/s.

## Ethics statement

The studies involving humans were approved by Ethics Committee of Zhejiang Cancer Hospital. The studies were conducted in accordance with the local legislation and institutional requirements. The ethics committee/institutional review board waived the requirement of written informed consent for participation from the participants or the participants’ legal guardians/next of kin because written informed consent for participation was not required from the participants in accordance with the national legislation and institutional requirements.

## Author contributions

XG: Conceptualization, Data curation, Funding acquisition, Investigation, Writing – original draft. HC: Conceptualization, Data curation, Methodology, Writing – original draft. YW: Data curation, Writing – review & editing. FX: Data curation, Writing – review & editing. AZ: Data curation, Writing – review & editing. YY: Conceptualization, Methodology, Supervision, Writing – review & editing. YG: Conceptualization, Funding acquisition, Project administration, Supervision, Writing – review & editing.

## References

[1] Yildirim Simsir I Cetinkalp S Kabalak T. Review of factors contributing to nodular goiter and thyroid carcinoma. Med Princ Pract. (2020) 29:1–5. 31542786 10.1159/000503575 PMC7024874

[2] Thakur S Daley B Klubo-Gwiezdzinska J. The role of an anti-diabetic drug metformin in the treatment of endocrine tumors. J Mol Endocrinol. (2019) 63:R17–r35. doi: 10.1530/JME-19-0083 31307011 PMC6938582

[3] He X Wu D Hu C Xu T Liu Y Liu C. Role of metformin in the treatment of patients with thyroid nodules and insulin resistance: A systematic review and meta-analysis. Thyroid. (2019) 29:359–67. doi: 10.1089/thy.2017.0707 30595105

[4] Kim HR Son M Huh SJ Moon SY Moon H Kang YW. Relationship between METS-IR and thyroid cancer incidence in Korea: a nationwide population-based study. Front Oncol. (2024) 14:1383864. doi: 10.3389/fonc.2024.1383864 38665956 PMC11044182

[5] Araque KA Gubbi S Klubo-Gwiezdzinska J. Updates on the management of thyroid cancer. Horm Metab Res. (2020) 52:562–77. 10.1055/a-1089-7870 PMC7415555 32040962

[6] Brose MS Nutting CM Jarzab B Elisei R Siena S Bastholt L. Sorafenib in radioactive iodine-refractory, locally advanced or metastatic differentiated thyroid cancer: a randomised, double-blind, phase 3 trial. Lancet. (2014) 384:319–28. doi: 10.1016/S0140-6736(14)60421-9 PMC4366116 24768112

[7] Seib CD Sosa JA. Evolving understanding of the epidemiology of thyroid cancer. Endocrinol Metab Clin North Am. (2019) 48:23–35. doi: 10.1016/j.ecl.2018.10.002 30717905

[8] Zhao J Zhang Q Yang Y Yao J Liao L Dong J. High prevalence of thyroid carcinoma in patients with insulin resistance: a meta-analysis of case-control studies. Aging (Albany NY). (2021) 13:22232–41. doi: 10.18632/aging.v13i18 PMC8507263 34550096

[9] Schlumberger M Brose M Elisei R Leboulleux S Luster M Pitoia F. Definition and management of radioactive iodine-refractory differentiated thyroid cancer. Lancet Diabetes Endocrinol. (2014) 2:356–8. 10.1016/S2213-8587(13)70215-8 24795243

[10] Mazzaferri EL Kloos RT. Clinical review 128: Current approaches to primary therapy for papillary and follicular thyroid cancer. J Clin Endocrinol Metab. (2001) 86:1447–63. doi: 10.1210/jcem.86.4.7407 11297567

[11] Alzahrani AS Alkhafaji D Tuli M Al-Hindi H Sadiq BB. Comparison of differentiated thyroid cancer in children and adolescents (≤20 years) with young adults. Clin Endocrinol (Oxf). (2016) 84:571–7. 10.1111/cen.12845 26118454

[12] Bal CS Kumar A Chandra P Dwivedi SN Mukhopadhyaya S. Is chest x-ray or high-resolution computed tomography scan of the chest sufficient investigation to detect pulmonary metastasis in pediatric differentiated thyroid cancer? Thyroid. (2004) 14:217–25. doi: 10.1089/105072504773297894 15072704

[13] Chesover AD Vali R Hemmati SH Wasserman JD. Lung metastasis in children with differentiated thyroid cancer: factors associated with diagnosis and outcomes of therapy. Thyroid. (2021) 31:50–60. 32517539 10.1089/thy.2020.0002

[14] Haugen BR Alexander EK Bible KC Doherty GM Mandel SJ Nikiforov YE. 2015 American thyroid association management guidelines for adult patients with thyroid nodules and differentiated thyroid cancer: the American thyroid association guidelines task force on thyroid nodules and differentiated thyroid cancer. Thyroid. (2016) 26:1–133. 26462967 10.1089/thy.2015.0020 PMC4739132

[15] Brown SR Hall A Buckley HL Flanagan L Gonzalez de Castro D Farnell K. Investigating the potential clinical benefit of Selumetinib in resensitising advanced iodine refractory differentiated thyroid cancer to radioiodine therapy (SEL-I-METRY): protocol for a multicentre UK single arm phase II trial. BMC Cancer. (2019) 19:582. doi: 10.1186/s12885-019-5541-4 31200667 PMC6567392

[16] Wadsley J Gregory R Flux G Newbold K Du Y Moss L. SELIMETRY-a multicentre I-131 dosimetry trial: a clinical perspective. Br J Radiol. (2017) 90:20160637. doi: 10.1259/bjr.20160637 28291381 PMC5605100

[17] Cabanillas ME McFadden DG Durante C. Thyroid cancer. Lancet. (2016) 388:2783–95. doi: 10.1016/S0140-6736(16)30172-6 27240885

[18] Van Nostrand D. The benefits and risks of I-131 therapy in patients with well-differentiated thyroid cancer. Thyroid. (2009) 19:1381–91. 10.1089/thy.2009.1611 20001720

[19] Sawka AM Thabane L Parlea L Ibrahim-Zada I Tsang RW Brierley JD. Second primary Malignancy risk after radioactive iodine treatment for thyroid cancer: a systematic review and meta-analysis. Thyroid. (2009) 19:451–7. 10.1089/thy.2008.0392 19281429

[20] Sugino K Nagahama M Kitagawa W Ohkuwa K Uruno T Matsuzu K. Distant metastasis in pediatric and adolescent differentiated thyroid cancer: clinical outcomes and risk factor analyses. J Clin Endocrinol Metab. (2020) 105. 10.1210/clinem/dgaa545 32813019

[21] Sun R Limkin EJ Vakalopoulou M Dercle L Champiat S Han SR. A radiomics approach to assess tumour-infiltrating CD8 cells and response to anti-PD-1 or anti-PD-L1 immunotherapy: an imaging biomarker, retrospective multicohort study. Lancet Oncol. (2018) 19:1180–91. doi: 10.1016/S1470-2045(18)30413-3 30120041

[22] Sabra MM Ghossein R Tuttle RM. Time course and predictors of structural disease progression in pulmonary metastases arising from follicular cell-derived thyroid cancer. Thyroid. (2016) 26:518–24. doi: 10.1089/thy.2015.0395 PMC5076482 26872102

[23] Sohn SY Kim HI Kim YN Kim TH Kim SW Chung JH. Prognostic indicators of outcomes in patients with lung metastases from differentiated thyroid carcinoma during long-term follow-up. Clin Endocrinol (Oxf). (2018) 88:318–26. doi: 10.1111/cen.13489 28972676

[24] Kim M Kim WG Park S Kwon H Jeon MJ Lee JJ. Initial size of metastatic lesions is best prognostic factor in patients with metastatic differentiated thyroid carcinoma confined to the lung. Thyroid. (2017) 27:49–58. doi: 10.1089/thy.2016.0347 27750021

[25] Maithel SK Ginsberg MS D'Amico F DeMatteo RP Allen PJ Fong Y. Natural history of patients with subcentimeter pulmonary nodules undergoing hepatic resection for metastatic colorectal cancer. J Am Coll Surg. (2010) 210:31–8. doi: 10.1016/j.jamcollsurg.2009.09.032 PMC4497784 20123329

[26] Alkurt EG Şahin F Tutan B Canal K Turhan VB. The relationship between papillary thyroid cancer and triglyceride/glucose index, which is an indicator of insulin resistance. Eur Rev Med Pharmacol Sci. (2022) 26:6114–20. 10.26355/eurrev_202209_29629 36111913

[27] Schlumberger M Leboulleux S. Current practice in patients with differentiated thyroid cancer. Nat Rev Endocrinol. (2021) 17:176–88. doi: 10.1038/s41574-020-00448-z 33339988

[28] Okamoto S Shiga T Uchiyama Y Manabe O Kobayashi K Yoshinaga K. Lung uptake on I-131 therapy and short-term outcome in patients with lung metastasis from differentiated thyroid cancer. Ann Nucl Med. (2014) 28:81–7. 10.1007/s12149-013-0781-x PMC3971446 24374647

[29] McDougall IR Davidson J Segall GM. Positron emission tomography of the thyroid, with an emphasis on thyroid cancer. Nucl Med Commun. (2001) 22:485–92. doi: 10.1097/00006231-200105000-00004 11388568

[30] Nakajo M Nakajo M Jinguji M Tani A Kajiya Y Tanabe H. Diagnosis of metastases from postoperative differentiated thyroid cancer: comparison between FDG and FLT PET/CT studies. Radiology. (2013) 267:891–901. doi: 10.1148/radiol.13121546 23468571

[31] Agate L Bianchi F Giorgetti A Sbragia P Bottici V Brozzi F. Detection of metastases from differentiated thyroid cancer by different imaging techniques (neck ultrasound, computed tomography and [18F]-FDG positron emission tomography) in patients with negative post-therapeutic ¹³¹I whole-body scan and detectable serum thyroglobulin levels. J Endocrinol Invest. (2014) 37:967–72. doi: 10.1007/s40618-014-0134-1 25070043

[32] Li C Wu Q Sun S. Radioactive iodine therapy in patients with thyroid carcinoma with distant metastases: A SEER-based study. Cancer Control. (2020) 27:1073274820914661. doi: 10.1177/1073274820914661 32292051 PMC7160783

[33] Perrier ND Brierley JD Tuttle RM. Differentiated and anaplastic thyroid carcinoma: Major changes in the American Joint Committee on Cancer eighth edition cancer staging manual. CA Cancer J Clin. (2018) 68:55–63. doi: 10.3322/caac.21439 29092098 PMC5766386

[34] Huang X Xia Q Huang Y Peng A Yang J. Age increased the cancer-specific mortality risk of thyroid cancer with lung metastasis. Clin Endocrinol (Oxf). (2022) 96:719–27. doi: 10.1111/cen.14675 34990026

[35] Chopra S Garg A Ballal S Bal CS. Lung metastases from differentiated thyroid carcinoma: prognostic factors related to remission and disease-free survival. Clin Endocrinol (Oxf). (2015) 82:445–52. doi: 10.1111/cen.12558 25040494

[36] Cho SW Choi HS Yeom GJ Lim JA Moon JH Park DJ. Long-term prognosis of differentiated thyroid cancer with lung metastasis in Korea and its prognostic factors. Thyroid. (2014) 24:277–86. 10.1089/thy.2012.0654 PMC3926138 23758653

[37] Zhao J Tian Y Jia Z Yao J Liao L Dong J. Abnormal glucose metabolism parameters and the aggressiveness of differentiated thyroid carcinoma: A hospital-based cross-section study in China. Front Endocrinol (Lausanne). (2022) 13:806349. 35299970 10.3389/fendo.2022.806349 PMC8921453

[38] Sánchez-García A Rodríguez-Gutiérrez R Mancillas-Adame L González-Nava V Díaz González-Colmenero A Solis RC. Diagnostic accuracy of the triglyceride and glucose index for insulin resistance: A systematic review. Int J Endocrinol. (2020) 2020:4678526. doi: 10.1155/2020/4678526 32256572 PMC7085845

[39] Chen ST Hsueh C Chiou WK Lin JD. Disease-specific mortality and secondary primary cancer in well-differentiated thyroid cancer with type 2 diabetes mellitus. PLoS One. (2013) 8:e55179. doi: 10.1371/journal.pone.0055179 23383099 PMC3561360

[40] Li LR Song JL Liu HQ Chen C. Metabolic syndrome and thyroid Cancer: risk, prognosis, and mechanism. Discovery Oncol. (2023) 14:23. 10.1007/s12672-022-00599-7 PMC9947216 36811728

