# Supplementary material for: Automatic prediction of non-iodine-avid status in lung metastases for radioactive I131 treatment in differentiated thyroid cancer patients
Source: Front Endocrinol (Lausanne). 2024 Jun 11;15:1429115. doi: 10.3389/fendo.2024.1429115 (PMC11201526; doi:10.3389/fendo.2024.1429115)
Supplement: Supplementary file 1 [file DataSheet_1.docx]

Supplementary Material A

**Detailed CT imaging protocols**

Preparation before scanning: patients were trained to hold their breath before scanning.

The position and mode of scanning: supine position, hands holding head, cross-sectional spiral scanning.

Scanning angle: 90 degrees right angle to the CT scanning bed.

Scanning range: from the tip of the lung to the bottom of the lung.

Scanners:

Ingenuity 64 (Philips Healthcare, Best, The Netherlands), Sensation 16 (Siemens Medical Solutions, Forchheim, Germany), Force (Siemens Medical Solutions, Forchheim, Germany), SOMATOM Definition Flash 64 (Siemens Medical Solutions, Forchheim, Germany); Revolution 256 (GE Healthcare, California, USA).

Field of view (FOV): 35cm*35cm*35cm-40cm*40cm*40cm, depending on the shape of the patient, the skin of the chest wall should be included.

Scanning parameters: 1mm-3mm slice thickness, slice interval, 1-3mm; matrix, 512 × 512, 100-120kV, automatic mA (100-300mA), 0.5-1.0s/r.

Reconstruction algorithm: high resolution algorithm.

Observation parameters: lung window width: 1600-2000 HU, lung window level: -800- -600 HU.
