# Supplementary material for: Automatic prediction of non-iodine-avid status in lung metastases for radioactive I131 treatment in differentiated thyroid cancer patients
Source: Front Endocrinol (Lausanne). 2024 Jun 11;15:1429115. doi: 10.3389/fendo.2024.1429115 (PMC11201526; doi:10.3389/fendo.2024.1429115)
Supplement: Supplementary file 2 [file DataSheet_2.docx]

Supplementary Material C

| Round | Dice-Loss | Dice-coefficient |
| --- | --- | --- |
| 0 | 0.974828319 | 0.025171681 |
| 1 | 0.90064906 | 0.09935094 |
| 2 | 0.816627931 | 0.183372069 |
| 3 | 0.795332848 | 0.204667152 |
| 4 | 0.814870115 | 0.185129885 |
| 5 | 0.846167014 | 0.153832986 |
| 6 | 0.813847061 | 0.186152939 |
| 7 | 0.666609697 | 0.333390303 |
| 8 | 0.668189037 | 0.331810963 |
| 9 | 0.71330173 | 0.28669827 |
| 10 | 0.668278717 | 0.331721283 |
| 11 | 0.64639847 | 0.35360153 |
| 12 | 0.638165139 | 0.361834861 |
| 13 | 0.689406473 | 0.310593527 |
| 14 | 0.666083491 | 0.333916509 |
| 15 | 0.631349729 | 0.368650271 |
| 16 | 0.647003045 | 0.352996955 |
| 17 | 0.588997987 | 0.411002013 |
| 18 | 0.512079448 | 0.487920552 |
| 19 | 0.481193113 | 0.518806887 |
| 20 | 0.435258116 | 0.564741884 |
| 21 | 0.464540647 | 0.535459353 |
| 22 | 0.555070111 | 0.444929889 |
| 23 | 0.540570758 | 0.459429242 |
| 24 | 0.502788184 | 0.497211816 |
| 25 | 0.442149128 | 0.557850872 |
| 26 | 0.439220637 | 0.560779363 |
| 27 | 0.474805513 | 0.525194487 |
| 28 | 0.512753864 | 0.487246136 |
| 29 | 0.519094391 | 0.480905609 |
| 30 | 0.478488259 | 0.521511741 |
| 31 | 0.438711677 | 0.561288323 |
| 32 | 0.46370107 | 0.53629893 |
| 33 | 0.500321968 | 0.499678032 |
| 34 | 0.449956307 | 0.550043693 |
| 35 | 0.473624477 | 0.526375523 |
| 36 | 0.461541193 | 0.538458807 |
| 37 | 0.42475111 | 0.57524889 |
| 38 | 0.442170465 | 0.557829535 |
| 39 | 0.412213118 | 0.587786882 |
| 40 | 0.349534599 | 0.650465401 |
| 41 | 0.385662941 | 0.614337059 |
| 42 | 0.40774379 | 0.59225621 |
| 43 | 0.323016601 | 0.676983399 |
| 44 | 0.319999313 | 0.680000687 |
| 45 | 0.352979203 | 0.647020797 |
| 46 | 0.323207224 | 0.676792776 |
| 47 | 0.327224825 | 0.672775175 |
| 48 | 0.349189945 | 0.650810055 |
| 49 | 0.354049262 | 0.645950738 |
| 50 | 0.354529398 | 0.645470602 |
| 51 | 0.367920322 | 0.632079678 |
| 52 | 0.344605777 | 0.655394223 |
| 53 | 0.323020599 | 0.676979401 |
| 54 | 0.328971044 | 0.671028956 |
| 55 | 0.305101522 | 0.694898478 |
| 56 | 0.295429541 | 0.704570459 |
| 57 | 0.307436929 | 0.692563071 |
| 58 | 0.301228347 | 0.698771653 |
| 59 | 0.306763964 | 0.693236036 |
| 60 | 0.370325281 | 0.629674719 |
| 61 | 0.342779297 | 0.657220703 |
| 62 | 0.281620158 | 0.718379842 |
| 63 | 0.275851452 | 0.724148548 |
| 64 | 0.288392098 | 0.711607902 |
| 65 | 0.306432833 | 0.693567167 |
| 66 | 0.275679432 | 0.724320568 |
| 67 | 0.300198462 | 0.699801538 |
| 68 | 0.330746882 | 0.669253118 |
| 69 | 0.302255385 | 0.697744615 |
| 70 | 0.298117222 | 0.701882778 |
| 71 | 0.266719132 | 0.733280868 |
| 72 | 0.246259026 | 0.753740974 |
| 73 | 0.290440484 | 0.709559516 |
| 74 | 0.291629006 | 0.708370994 |
| 75 | 0.254019655 | 0.745980345 |
| 76 | 0.285811035 | 0.714188965 |
| 77 | 0.301838832 | 0.698161168 |
| 78 | 0.296001201 | 0.703998799 |
| 79 | 0.310693042 | 0.689306958 |
| 80 | 0.278638217 | 0.721361783 |
| 81 | 0.244338749 | 0.755661251 |
| 82 | 0.266305072 | 0.733694928 |
| 83 | 0.286641955 | 0.713358045 |
| 84 | 0.290280189 | 0.709719811 |
| 85 | 0.26334352 | 0.73665648 |
| 86 | 0.22324745 | 0.77675255 |
| 87 | 0.22418301 | 0.77581699 |
| 88 | 0.248915738 | 0.751084262 |
| 89 | 0.257972656 | 0.742027344 |
| 90 | 0.246047982 | 0.753952018 |
| 91 | 0.232184034 | 0.767815966 |
| 92 | 0.222464846 | 0.777535154 |
| 93 | 0.231361937 | 0.768638063 |
| 94 | 0.255014539 | 0.744985461 |
| 95 | 0.258422993 | 0.741577007 |
| 96 | 0.268017882 | 0.731982118 |
| 97 | 0.302459136 | 0.697540864 |
| 98 | 0.271526256 | 0.728473744 |
| 99 | 0.255358545 | 0.744641455 |
| 100 | 0.267730053 | 0.732269947 |
| 101 | 0.25855159 | 0.74144841 |
| 102 | 0.261297112 | 0.738702888 |
| 103 | 0.234296401 | 0.765703599 |
| 104 | 0.218942719 | 0.781057281 |
| 105 | 0.246937853 | 0.753062147 |
| 106 | 0.262169255 | 0.737830745 |
| 107 | 0.267623147 | 0.732376853 |
| 108 | 0.279159871 | 0.720840129 |
| 109 | 0.268196205 | 0.731803795 |
| 110 | 0.283237305 | 0.716762695 |
| 111 | 0.307966711 | 0.692033289 |
| 112 | 0.267736985 | 0.732263015 |
| 113 | 0.242297796 | 0.757702204 |
| 114 | 0.227826127 | 0.772173873 |
| 115 | 0.222519215 | 0.777480785 |
| 116 | 0.255856248 | 0.744143752 |
| 117 | 0.264633499 | 0.735366501 |
| 118 | 0.266567453 | 0.733432547 |
| 119 | 0.280961237 | 0.719038763 |
| 120 | 0.258998584 | 0.741001416 |
| 121 | 0.223005598 | 0.776994402 |
| 122 | 0.223924011 | 0.776075989 |
| 123 | 0.229567804 | 0.770432196 |
| 124 | 0.250377671 | 0.749622329 |
| 125 | 0.244132809 | 0.755867191 |
| 126 | 0.227418752 | 0.772581248 |
| 127 | 0.275903765 | 0.724096235 |
| 128 | 0.296719594 | 0.703280406 |
| 129 | 0.251996569 | 0.748003431 |
| 130 | 0.228007288 | 0.771992712 |
| 131 | 0.250106787 | 0.749893213 |
| 132 | 0.265338523 | 0.734661477 |
| 133 | 0.244260433 | 0.755739567 |
| 134 | 0.229691127 | 0.770308873 |
| 135 | 0.261710296 | 0.738289704 |
| 136 | 0.2814113 | 0.7185887 |
| 137 | 0.246221727 | 0.753778273 |
| 138 | 0.201959677 | 0.798040323 |
| 139 | 0.20219286 | 0.79780714 |
| 140 | 0.209537881 | 0.790462119 |
| 141 | 0.193665059 | 0.806334941 |
| 142 | 0.184526168 | 0.815473832 |
| 143 | 0.22129109 | 0.77870891 |
| 144 | 0.248004664 | 0.751995336 |
| 145 | 0.228827412 | 0.771172588 |
| 146 | 0.209890172 | 0.790109828 |
| 147 | 0.248566227 | 0.751433773 |
| 148 | 0.254997654 | 0.745002346 |
| 149 | 0.203997485 | 0.796002515 |
| 150 | 0.199364038 | 0.800635962 |
| 151 | 0.223532185 | 0.776467815 |
| 152 | 0.275489431 | 0.724510569 |
| 153 | 0.24537081 | 0.75462919 |
| 154 | 0.221455098 | 0.778544902 |
| 155 | 0.231992676 | 0.768007324 |
| 156 | 0.201390386 | 0.798609614 |
| 157 | 0.217300575 | 0.782699425 |
| 158 | 0.260642109 | 0.739357891 |
| 159 | 0.292934601 | 0.707065399 |
| 160 | 0.247170388 | 0.752829612 |
| 161 | 0.187874019 | 0.812125981 |
| 162 | 0.19030192 | 0.80969808 |
| 163 | 0.19831042 | 0.80168958 |
| 164 | 0.227890265 | 0.772109735 |
| 165 | 0.276270257 | 0.723729743 |
| 166 | 0.257355441 | 0.742644559 |
| 167 | 0.218361984 | 0.781638016 |
| 168 | 0.246385651 | 0.753614349 |
| 169 | 0.229844587 | 0.770155413 |
| 170 | 0.173910802 | 0.826089198 |
| 171 | 0.21542257 | 0.78457743 |
| 172 | 0.236927504 | 0.763072496 |
| 173 | 0.195201921 | 0.804798079 |
| 174 | 0.1943844 | 0.8056156 |
| 175 | 0.214239937 | 0.785760063 |
| 176 | 0.199893062 | 0.800106938 |
| 177 | 0.182104381 | 0.817895619 |
| 178 | 0.182109732 | 0.817890268 |
| 179 | 0.230909027 | 0.769090973 |
| 180 | 0.235159487 | 0.764840513 |
| 181 | 0.188895097 | 0.811104903 |
| 182 | 0.191716332 | 0.808283668 |
| 183 | 0.186949095 | 0.813050905 |
| 184 | 0.217291357 | 0.782708643 |
| 185 | 0.249000077 | 0.750999923 |
| 186 | 0.234605103 | 0.765394897 |
| 187 | 0.21399507 | 0.78600493 |
| 188 | 0.23464408 | 0.76535592 |
| 189 | 0.233973532 | 0.766026468 |
| 190 | 0.213212603 | 0.786787397 |
| 191 | 0.197907891 | 0.802092109 |
| 192 | 0.17181665 | 0.82818335 |
| 193 | 0.181889864 | 0.818110136 |
| 194 | 0.199319703 | 0.800680297 |
| 195 | 0.235993031 | 0.764006969 |
| 196 | 0.230934977 | 0.769065023 |
| 197 | 0.222951774 | 0.777048226 |
| 198 | 0.22617413 | 0.77382587 |
| 199 | 0.195597542 | 0.804402458 |
| 200 | 0.21047832 | 0.78952168 |
| 201 | 0.203980159 | 0.796019841 |
| 202 | 0.199717015 | 0.800282985 |
| 203 | 0.20425638 | 0.79574362 |
| 204 | 0.194234109 | 0.805765891 |
| 205 | 0.209043895 | 0.790956105 |
| 206 | 0.203687096 | 0.796312904 |
| 207 | 0.257901199 | 0.742098801 |
| 208 | 0.270784554 | 0.729215446 |
| 209 | 0.197069839 | 0.802930161 |
| 210 | 0.188776133 | 0.811223867 |
| 211 | 0.198999785 | 0.801000215 |
| 212 | 0.185620498 | 0.814379502 |
| 213 | 0.19206506 | 0.80793494 |
| 214 | 0.207645558 | 0.792354442 |
| 215 | 0.195780333 | 0.804219667 |
| 216 | 0.214646303 | 0.785353697 |
| 217 | 0.191089149 | 0.808910851 |
| 218 | 0.189532931 | 0.810467069 |
| 219 | 0.180155573 | 0.819844427 |
| 220 | 0.182136278 | 0.817863722 |
| 221 | 0.209925414 | 0.790074586 |
| 222 | 0.211933249 | 0.788066751 |
| 223 | 0.222471461 | 0.777528539 |
| 224 | 0.218624494 | 0.781375506 |
| 225 | 0.195048722 | 0.804951278 |
| 226 | 0.189667713 | 0.810332287 |
| 227 | 0.200256431 | 0.799743569 |
| 228 | 0.207985047 | 0.792014953 |
| 229 | 0.204631966 | 0.795368034 |
| 230 | 0.207016703 | 0.792983297 |
| 231 | 0.191684668 | 0.808315332 |
| 232 | 0.183467047 | 0.816532953 |
| 233 | 0.18457807 | 0.81542193 |
| 234 | 0.185029445 | 0.814970555 |
| 235 | 0.209317527 | 0.790682473 |
| 236 | 0.216399497 | 0.783600503 |
| 237 | 0.217282829 | 0.782717171 |
| 238 | 0.241467385 | 0.758532615 |
| 239 | 0.216195336 | 0.783804664 |
| 240 | 0.229205287 | 0.770794713 |
| 241 | 0.244101872 | 0.755898128 |
| 242 | 0.22982528 | 0.77017472 |
| 243 | 0.237311193 | 0.762688807 |
| 244 | 0.212911099 | 0.787088901 |
| 245 | 0.197235048 | 0.802764952 |
| 246 | 0.18555136 | 0.81444864 |
| 247 | 0.17614503 | 0.82385497 |
| 248 | 0.185278544 | 0.814721456 |
| 249 | 0.190158222 | 0.809841778 |
| 250 | 0.191063382 | 0.808936618 |
| 251 | 0.189801448 | 0.810198552 |
| 252 | 0.188088332 | 0.811911668 |
| 253 | 0.204198302 | 0.795801698 |
| 254 | 0.201242759 | 0.798757241 |
| 255 | 0.205957785 | 0.794042215 |
| 256 | 0.209474444 | 0.790525556 |
| 257 | 0.191446423 | 0.808553577 |
| 258 | 0.191102109 | 0.808897891 |
| 259 | 0.18864903 | 0.81135097 |
| 260 | 0.186508578 | 0.813491422 |
| 261 | 0.189784566 | 0.810215434 |
| 262 | 0.202487324 | 0.797512676 |
| 263 | 0.198396353 | 0.801603647 |
| 264 | 0.190052822 | 0.809947178 |
| 265 | 0.194278831 | 0.805721169 |
| 266 | 0.185224938 | 0.814775062 |
| 267 | 0.184024026 | 0.815975974 |
| 268 | 0.194076846 | 0.805923154 |
| 269 | 0.202385992 | 0.797614008 |
| 270 | 0.203995559 | 0.796004441 |
| 271 | 0.209673085 | 0.790326915 |
| 272 | 0.208586651 | 0.791413349 |
| 273 | 0.190558289 | 0.809441711 |
| 274 | 0.205207711 | 0.794792289 |
| 275 | 0.203021646 | 0.796978354 |
| 276 | 0.202261055 | 0.797738945 |
| 277 | 0.210977483 | 0.789022517 |
| 278 | 0.199019274 | 0.800980726 |
| 279 | 0.213232453 | 0.786767547 |
| 280 | 0.207461914 | 0.792538086 |
| 281 | 0.196239926 | 0.803760074 |
| 282 | 0.189734143 | 0.810265857 |
| 283 | 0.168782974 | 0.831217026 |
| 284 | 0.171284638 | 0.828715362 |
| 285 | 0.171214175 | 0.828785825 |
| 286 | 0.170090156 | 0.829909844 |
| 287 | 0.189056531 | 0.810943469 |
| 288 | 0.18935925 | 0.81064075 |
| 289 | 0.187623503 | 0.812376497 |
| 290 | 0.192571689 | 0.807428311 |
| 291 | 0.181836539 | 0.818163461 |
| 292 | 0.184313463 | 0.815686537 |
| 293 | 0.189217996 | 0.810782004 |
| 294 | 0.185938951 | 0.814061049 |
| 295 | 0.18553635 | 0.81446365 |
| 296 | 0.187641988 | 0.812358012 |
| 297 | 0.185407685 | 0.814592315 |
| 298 | 0.185247434 | 0.814752566 |
| 299 | 0.179604771 | 0.820395229 |
| 300 | 0.171370872 | 0.828629128 |
| 301 | 0.178877274 | 0.821122726 |
| 302 | 0.179384585 | 0.820615415 |
| 303 | 0.178364042 | 0.821635958 |
| 304 | 0.181236463 | 0.818763537 |
| 305 | 0.185210969 | 0.814789031 |
| 306 | 0.187386783 | 0.812613217 |
| 307 | 0.178471496 | 0.821528504 |
| 308 | 0.190417736 | 0.809582264 |
| 309 | 0.19061586 | 0.80938414 |
| 310 | 0.187288469 | 0.812711531 |
| 311 | 0.197682652 | 0.802317348 |
| 312 | 0.187887045 | 0.812112955 |
| 313 | 0.188370826 | 0.811629174 |
| 314 | 0.188539199 | 0.811460801 |
| 315 | 0.186328628 | 0.813671372 |
| 316 | 0.186937375 | 0.813062625 |
| 317 | 0.182623213 | 0.817376787 |
| 318 | 0.182151465 | 0.817848535 |
| 319 | 0.17911974 | 0.82088026 |
