# Supplementary material for: Automatic prediction of non-iodine-avid status in lung metastases for radioactive I131 treatment in differentiated thyroid cancer patients
Source: Front Endocrinol (Lausanne). 2024 Jun 11;15:1429115. doi: 10.3389/fendo.2024.1429115 (PMC11201526; doi:10.3389/fendo.2024.1429115)

Supplementary Material D

1. The CT images of a RAI-avid LM and the heat maps of the features for the SE Net deep learning model:


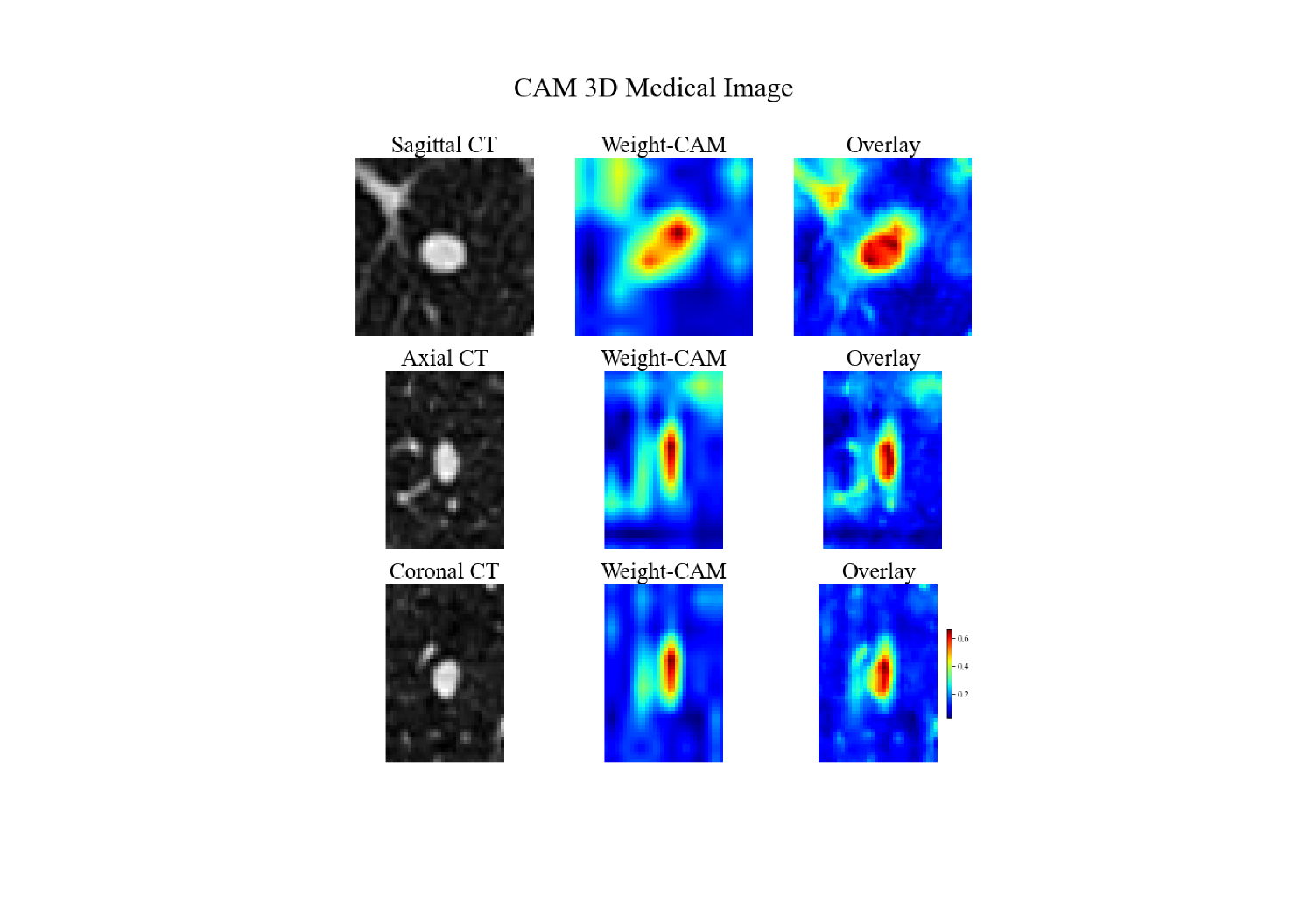


1. The CT images of a RAI-refractory LM and the heat maps of the features for the SE Net deep learning model:


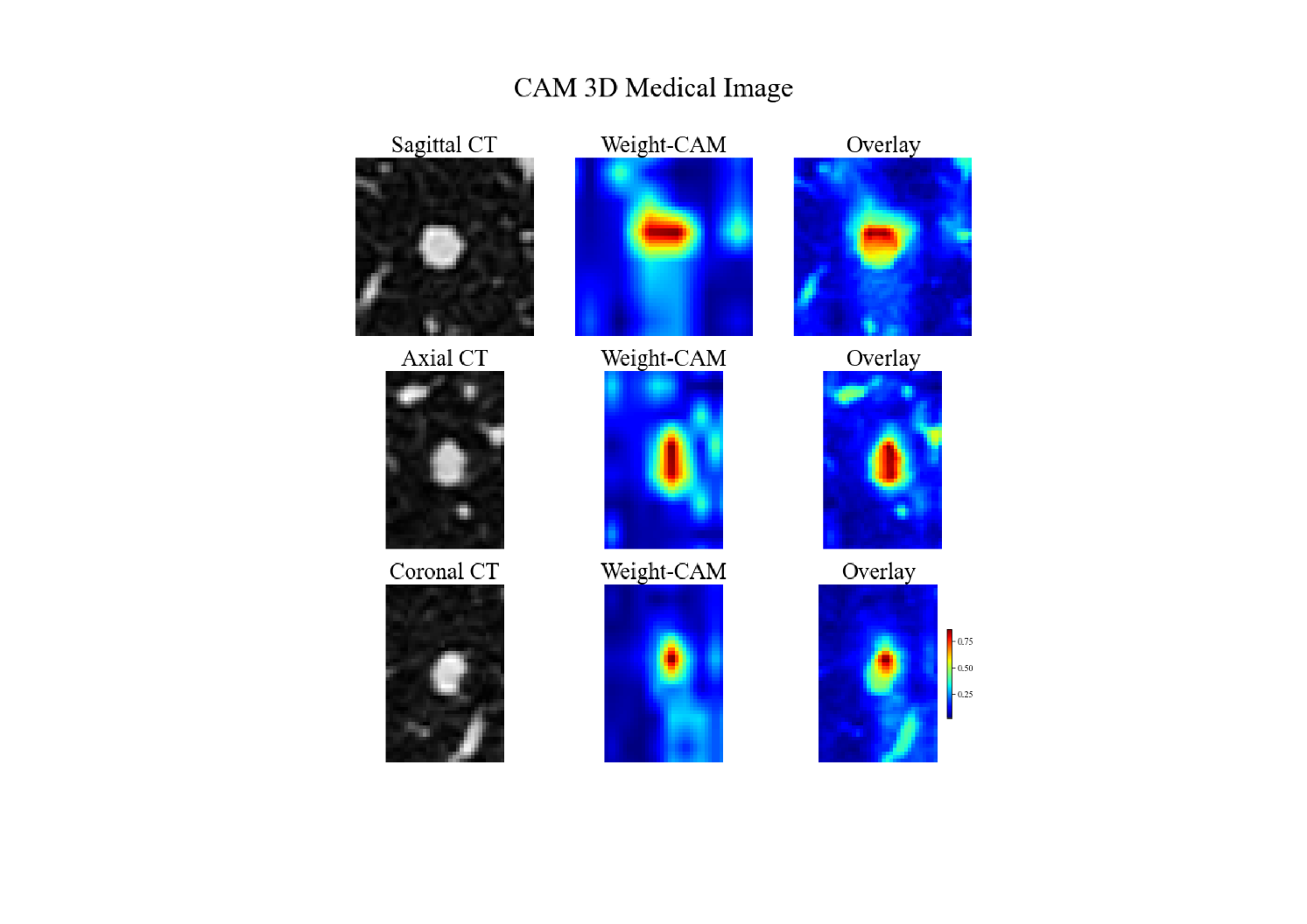

Supplement: Supplementary file 3 [file DataSheet_3.docx]
