# Supplementary figures and images for: Automatic prediction of non-iodine-avid status in lung metastases for radioactive I131 treatment in differentiated thyroid cancer patients
Source: Front Endocrinol (Lausanne). 2024 Jun 11;15:1429115. doi: 10.3389/fendo.2024.1429115 (PMC11201526; doi:10.3389/fendo.2024.1429115)

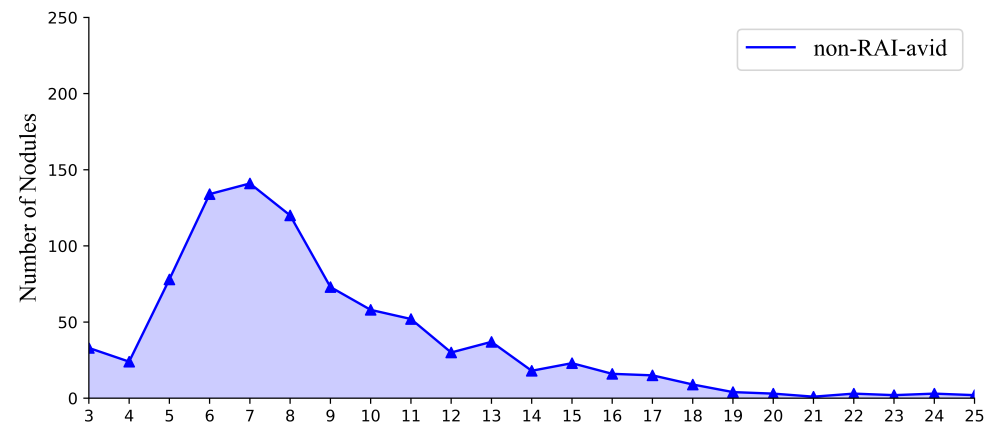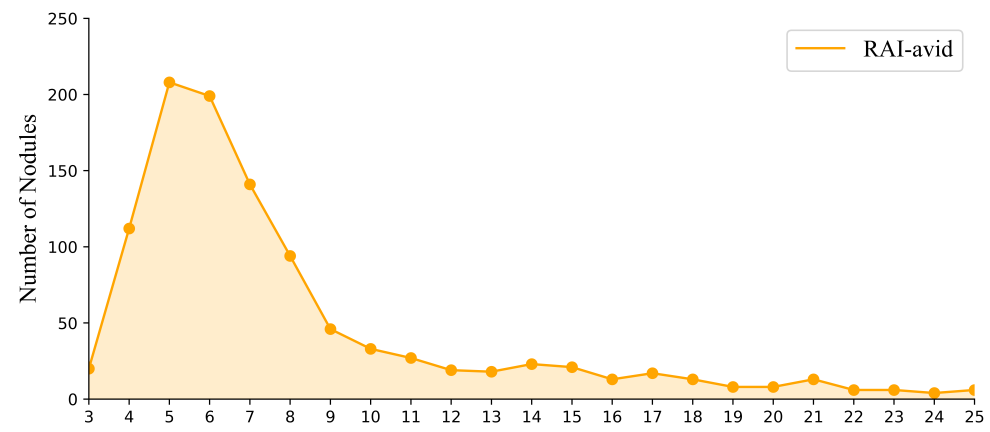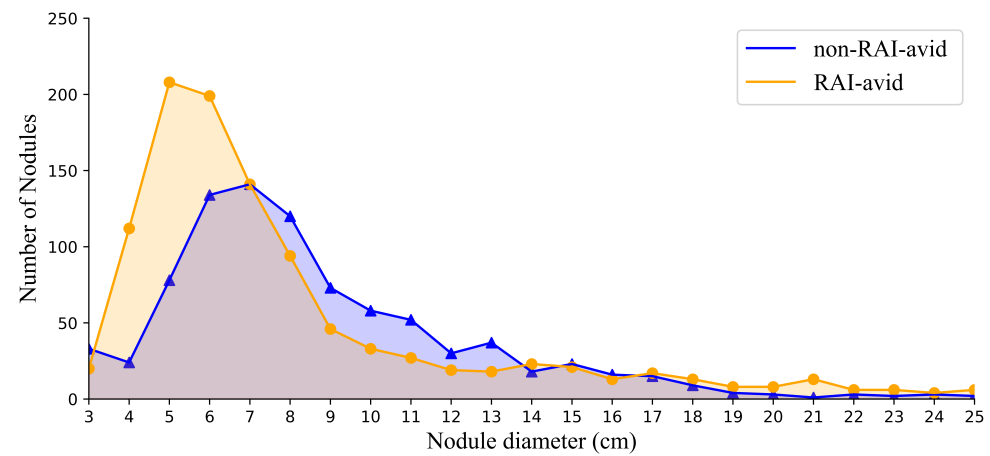

Supplement: Supplementary file 4 [file Image_1.pdf]
